# Supplementary material for: Variations of Secondary Metabolites among Natural Populations of Sub-Antarctic Ranunculus Species Suggest Functional Redundancy and Versatility
Source: Plants (Basel). 2019 Jul 19;8(7):234. doi: 10.3390/plants8070234 (PMC6681328; doi:10.3390/plants8070234)
Supplement: Supplementary file 1 [file plants-08-00234-s001.zip › plants-523601--/Labarrere_et_al.Supplementary Figures S1 to S4.docx]

**Figure S1**. Relationships between individual amines and individual environmental variables from simple regression analyses, within and across regions. Sign of significant p-values (<0.05) after sequential Bonferroni's correction are presented. In *R. biternatus*, sample size (N) = 58; in *R. pseudotrullifolius* N=57; in *R. moseleyi* N=46. Abbreviation: Agm: Agmatine; Put: Putrescine; Spm: Spermine; Spd: Spermidine; Cad: Cadaverine; DAP: 1,3-diaminopropane; Dop: Dopamine; Ser: Serotonin; Tyr: Tyramine; Oct: Octopamine; N1Ac.Spm: N^1^-acetylspermine; N8Ac.Spd: N^8^-acetylspermidine; Try: Tryptamine; 3M4OHPhe: 3-methoxy-4-hydroxy phenylethylamine (See Table 3 and Fig. 2 for increasingly complete multivariate relationships between environmental variables and compounds).

**Figure S2**. Relationships between individual quercetins and individual environmental variables from simple regression analyses, within and across regions. Sign of significant p-values (<0.05) after sequential Bonferroni's correction are presented. In *R. biternatus*, sample size (N) = 52; in *R. pseudotrullifolius* N=48; in *R. moseleyi* N=26. Abbreviation: Q-3GL: Quercetin 3-diglucoside-7-glucoside; Q-3GL+caf: Quercetin 3-(caffeyl-glucosyl)glucoside-7-glucoside; Q-3GL+Fer: quercetin 3-(ferulyl-glucosyl)glucoside-7-glucoside; Q-2GL+Xyl+Caf: quercetin 3-(caffeyl-xylosyl)glucoside-7-glucoside; Q-2GL-Xyl+Fer: Quercetin 3-(ferulyl-xylosyl)glucoside-7-glucoside; Q-2GL-Xyl : Quercetin 3-xylosylglucoside-7-glucoside; Q-GL-Xyl : Quercetin 3-xylosylglucoside; Q-2GL : Quercetin 3-diglucoside; IQC: Isoquercitrin. (See Table 3 for multivariate relationships between environmental variables and compounds).

**Figure S3.** Relationships between individual amines and individual traits from simple regression analyses, within and across regions. Sign of significant p-values (<0.05) after sequential Bonferroni's correction are presented. In *R. biternatus*, sample size (N) = 58; in *R. pseudotrullifolius* N=57; in *R. moseleyi* N=46. Abbreviation: Agm: Agmatine; Put: Putrescine; Spm: Spermine; Spd: Spermidine; Cad: Cadaverine; DAP: 1,3-diaminopropane; Dop: Dopamine; Ser: Serotonin; Tyr: Tyramine; Oct: Octopamine; N1Ac-Spm: N1-acetylspermine; N8Ac-Spd: N8-acetylspermidine; Try: Tryptamine; 3M4OHPhe: 3-methoxy-4-hydroxy phenylethylamine. (See Table 4 and Fig. S5 for increasingly complete multivariate relationships between traits and compounds).

**Figure S4.** Relationships between individual quercetins and individual traits from simple regression analyses, within and across regions. Sign of significant p-values (<0.05) after sequential Bonferroni's correction are presented. In *R. biternatus*, sample size (N) = 52; in *R. pseudotrullifolius* N=48; in *R. moseleyi* N=26. Abbreviation: Q-3GL: Quercetin 3-diglucoside-7-glucoside; Q-3GL+caf: Quercetin 3-(caffeyl-glucosyl)glucoside-7-glucoside; Q-3GL+Fer: quercetin 3-(ferulyl-glucosyl)glucoside-7-glucoside; Q-2GL+Xyl+Caf: quercetin 3-(caffeyl-xylosyl)glucoside-7-glucoside; Q-2GL-Xyl+Fer: Quercetin 3-(ferulyl-xylosyl)glucoside-7-glucoside; Q-2GL-Xyl : Quercetin 3-xylosylglucoside-7-glucoside; Q-GL-Xyl : Quercetin 3-xylosylglucoside; Q-2GL : Quercetin 3-diglucoside; IQC: Isoquercitrin. (See Table 4 for multivariate relationships between traits and compounds).
