# Supplementary material for: Variations of Secondary Metabolites among Natural Populations of Sub-Antarctic Ranunculus Species Suggest Functional Redundancy and Versatility
Source: Plants (Basel). 2019 Jul 19;8(7):234. doi: 10.3390/plants8070234 (PMC6681328; doi:10.3390/plants8070234)
Supplement: Supplementary file 1 [file plants-08-00234-s001.zip › plants-523601--/Labarrere_et_al.Supplementary Tables S1 and S2.docx]

Supplementary tables

**Table S1.** Relationship between total levels of amines or quercetins and environmental conditions. Simple regression analyses, indicated are adjusted r squares and p-values after sequential Bonferroni's correction. Significant p-values are indicated in bold, signs of significant relationships are indicated. Amine analyses: *R. biternatus*, sample size (N) = 58; *R. pseudotrullifolius* N=57; *R. moseleyi* N=46. Quercetin analyses: *R. biternatus*, sample size (N) = 52; *R. pseudotrullifolius* N=48; *R. moseleyi* N=26.

**Table S2.** Relationship between traits and total levels of amines or quercetins. Simple regression analyses, indicated are adjusted r squares and p-values after sequential Bonferroni's correction. Significant p-values are indicated in bold, signs of significant relationships are indicated. Amine analyses: *R. biternatus*, sample size (N) = 58; *R. pseudotrullifolius* N=57; *R. moseleyi* N=46. Quercetin analyses: *R. biternatus*, sample size (N) = 52; *R. pseudotrullifolius* N=48; *R. moseleyi* N=26.
